# Gender-specific perception of socio-psychological risks during the Covid-19 pandemic

**DOI:** 10.1192/j.eurpsy.2022.1373

**Published:** 2022-09-01

**Authors:** E. Belinskaya, O. Tihomandritskaya, E. Dubovskaya, T. Popova

**Affiliations:** Lomonosov Moscow State University, Faculty Of Psychology, Moscow, Russian Federation

**Keywords:** Adaptation, Gender psychology, Adaptation, gender psychology

## Abstract

**Introduction:**

The article is devoted to the study of adaptation to the covid-19 pandemic conditions in Russian men and women in the first part of 2020. Respondents assessed the degree of potential danger of various types of social threats caused by the pandemic, as well as the level of their adaptation to changed living conditions during the first lockdown.

**Objectives:**

The purpose of this study was to identify key differences in adaptation to pandemic conditions in groups of men and women.

**Methods:**

The author’s methodology was developed to assess the level of adaptation to COVID-19. The questionnaire included 6 scales on different aspects of life during the lockdown (for example, physical and emotional state, communication, employment during the pandemic, etc.). The sample was N = 80 (residents of Russia age 18-37).

**Results:**

Men significantly higher estimated the potential danger of an epidemic threat to themselves than women. The level of adaptation to conditions caused by the COVID-19 pandemic also differed in two gender groups according to t-test on the scale “Employment in a pandemic”: men were better adapted to work or study in conditions caused by the COVID-19 pandemic than women.

**Conclusions:**

This can be interpreted from the point of view of the stereotype existing in Russian culture which postulates the performance of household duties and child care is more perceived as a woman’s responsibility. The adaptation of women to remote work during the lockdown was forced to be combined with an increased workload in the family and household sphere.

**Disclosure:**

No significant relationships.

